# Supplementary figures and images for: The Mycotoxin Beauvericin Exhibits Immunostimulatory Effects on Dendritic Cells via Activating the TLR4 Signaling Pathway
Source: Front Immunol. 2022 Apr 8;13:856230. doi: 10.3389/fimmu.2022.856230 (PMC9024221; doi:10.3389/fimmu.2022.856230)

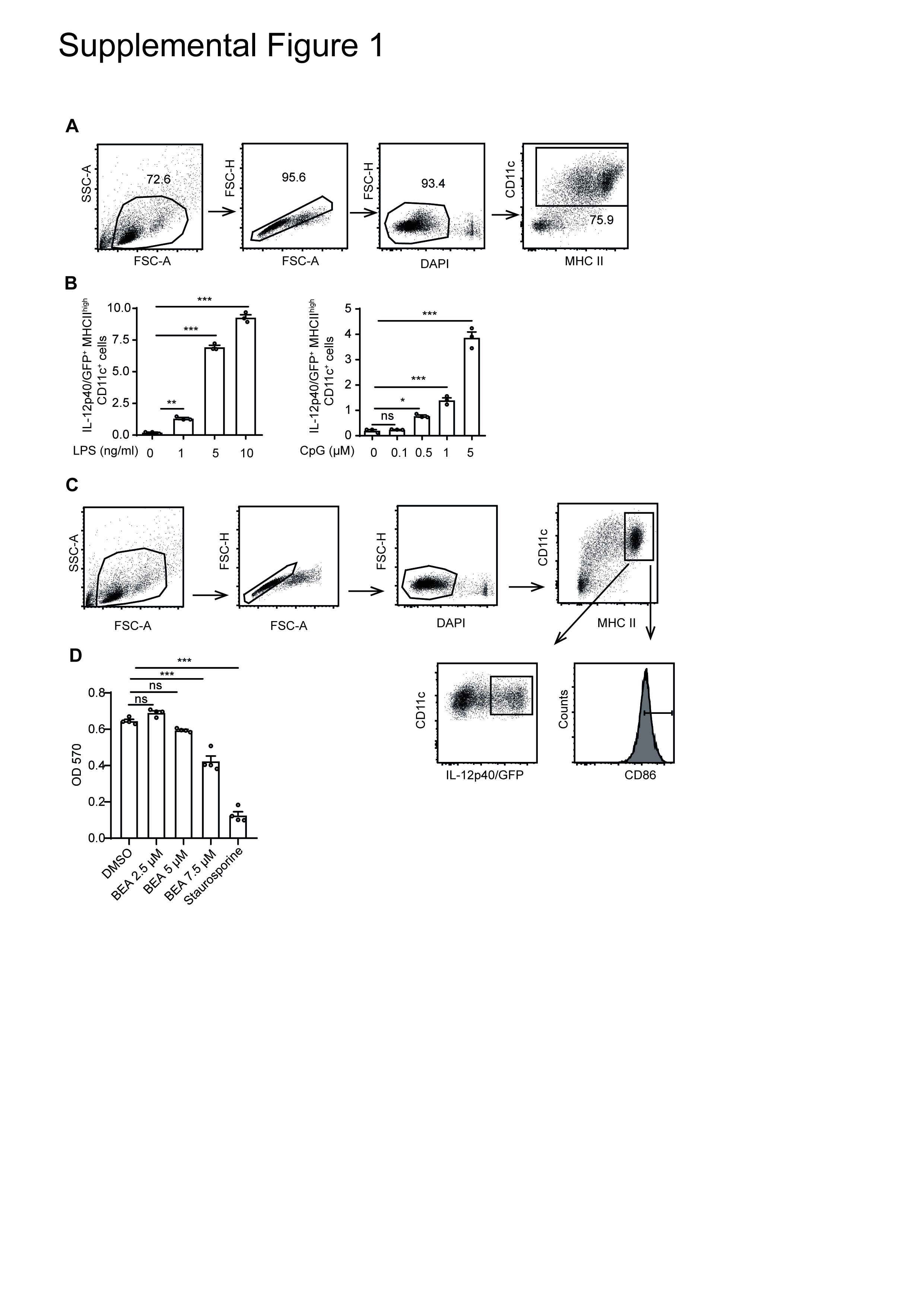

Supplement: Supplementary Figure 1 — Gating strategies and establishment of culture conditions for the analysis of effects of BEA on BMDCs. (A) Purity of BMDCs. Arrows indicate that BMDCs were sequentially gated from single cells, live cells (DAPI-) and CD11c+ cells. (B) Optimization of LPS and CpG treatment on IL-12p40/GFP production by BMDCs. 5x10^5^ BMDCs from IL-12p40/GFP reporter mice were stimulated with indicated concentration of LPS or CpG2216 for 16 hours. IL-12p40/GFP expression by BMDCs were detected by flow cytometry. Data shown are from one experiment using cells from 3 mice per group (n=3). Data are presented as mean ± SEM and analyzed by one-way ANOVA with Dunnett’s multiple comparisons test. *p<0.05, **p<0.01, ***p<0.001, ns, not significant. (C) Representative gating strategy for IL-12p40/GFP and CD86 expression in BMDCs. Arrows indicate that BMDCs were sequentially gated from single cells, live cells (DAPI-). Gated MHC IIhigh CD11c+ BMDCs were analyzed for expression of IL-12p40/GFP or CD86. (D) Analysis of potential cytotoxic effects of BEA on BMDCs. 8x10^4^ BMDCs were stimulated with the indicated concentration of BEA, DMSO or staurosporine for 16 hours. Cell viability was analyzed by using an MTT assay and measuring the absorbance of OD570. Results shown are representative of two independent experiments using cells from 3 mice per group (n=3). Data are presented as means ± SEM. Significance was analyzed by one-way ANOVA with Dunnett’s multiple comparisons test. ***p<0.001, ns, no significance. [file Image_1.tif]

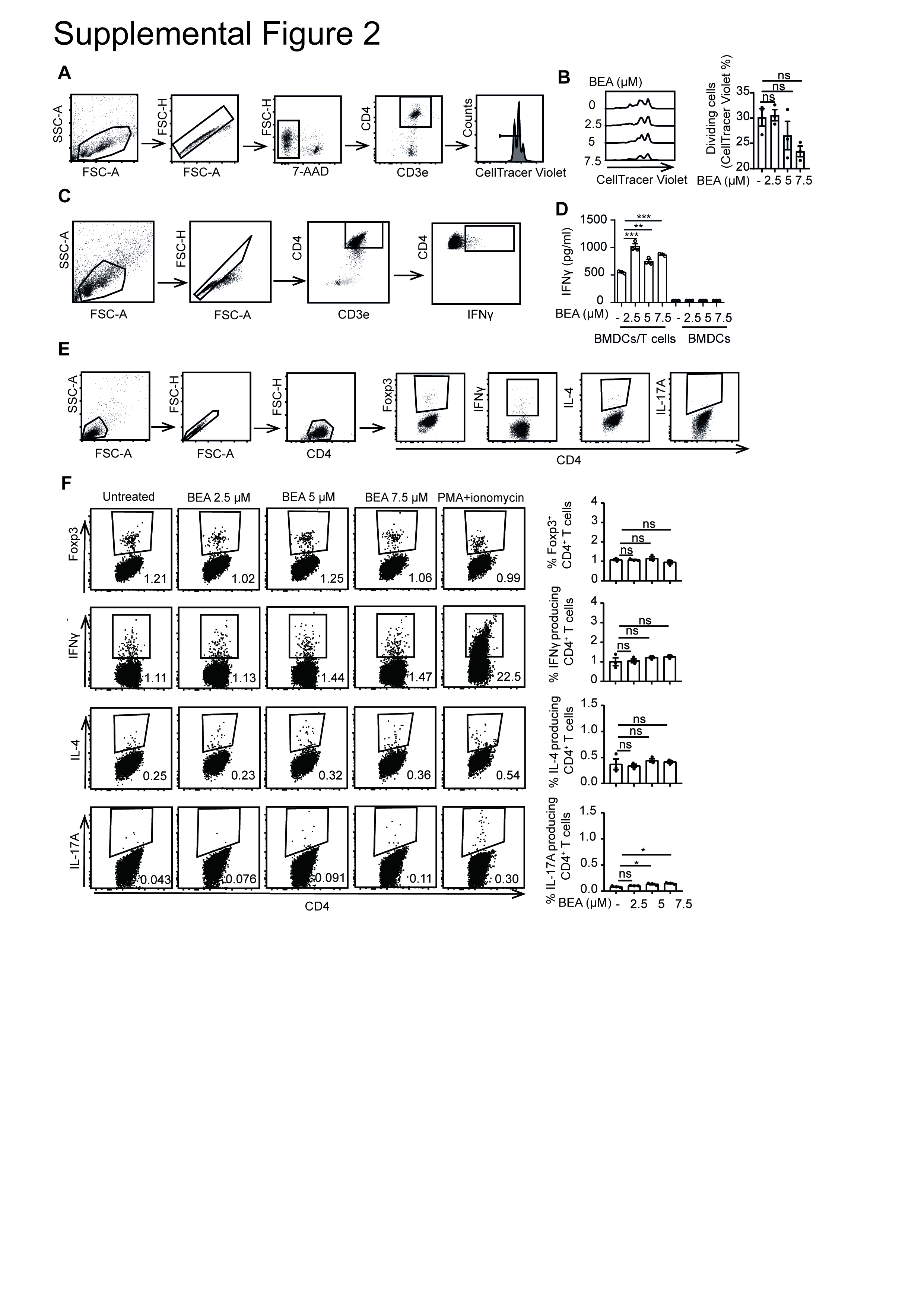

Supplement: Supplementary Figure 2 — Effects of BEA-treated BMDCs or BEA on T cell proliferation and differentiation. (A) Representative gating strategy for T cell proliferation. Arrows indicate that CD4 T cells were sequentially gated from single cells and live cells (7-AAD-). Gated CD4+ CD3e+ T cells were analyzed for T cell proliferation. (B) Direct impact of BEA on T cell proliferation. 105 naïve OT II CD4+ T cells labelled with CellTracer Violet were stimulated with or without indicated concentration of BEA in the presence of IL-2. T cell proliferation was detected at day 3 by flow cytometry. Data shown are from one experiment using cells from 3 mice per group (n=3). Data are presented as mean ± SEM and analyzed by one-way ANOVA with Dunnett’s multiple comparisons test. ns: not significant. (C) Representative gating strategy for T cell IFNγ production. Arrows indicate that CD4+ T cells were gated from single cells. Gated CD4+ CD3e+ T cells were analyzed for IFNγ production. (D) IFNγ concentrations in supernatants of BMDCs co-cultured T cells or BMDCs. 105 naïve OT-II CD4+ T cells were cultured with 104 untreated or BEA-treated BMDCs for 5 days in the presence of OVA peptide. IFNγ production in the supernatant was detected by ELISA. Results shown are from one experiment using cells from 3 mice per group (n=3). Data are presented as mean ± SEM and analyzed by one-way ANOVA with Dunnett’s multiple comparisons test. **p<0.01, ***p<0.001. (E) Representative gating strategy for combined analysis of T helper cell differentiation associated transcription factors and cytokines. Arrows indicate that CD4+ T cells were gated from single cells. Gated CD4+ T cells were analyzed for Foxp3 expression or production of IFNγ, IL-4, and IL-17A. (F) Expression of Foxp3, IFNγ, IL-4 and IL-17A in T cells co-cultured with untreated or BEA treated BMDCs. 105 naïve OT II CD4+ T cells were cultured with 104 untreated or BEA-treated BMDCs in the presence of OVA peptide. Expression of Foxp3, IFNγ, IL-4 and IL-17A were detecte [file Image_2.tif]

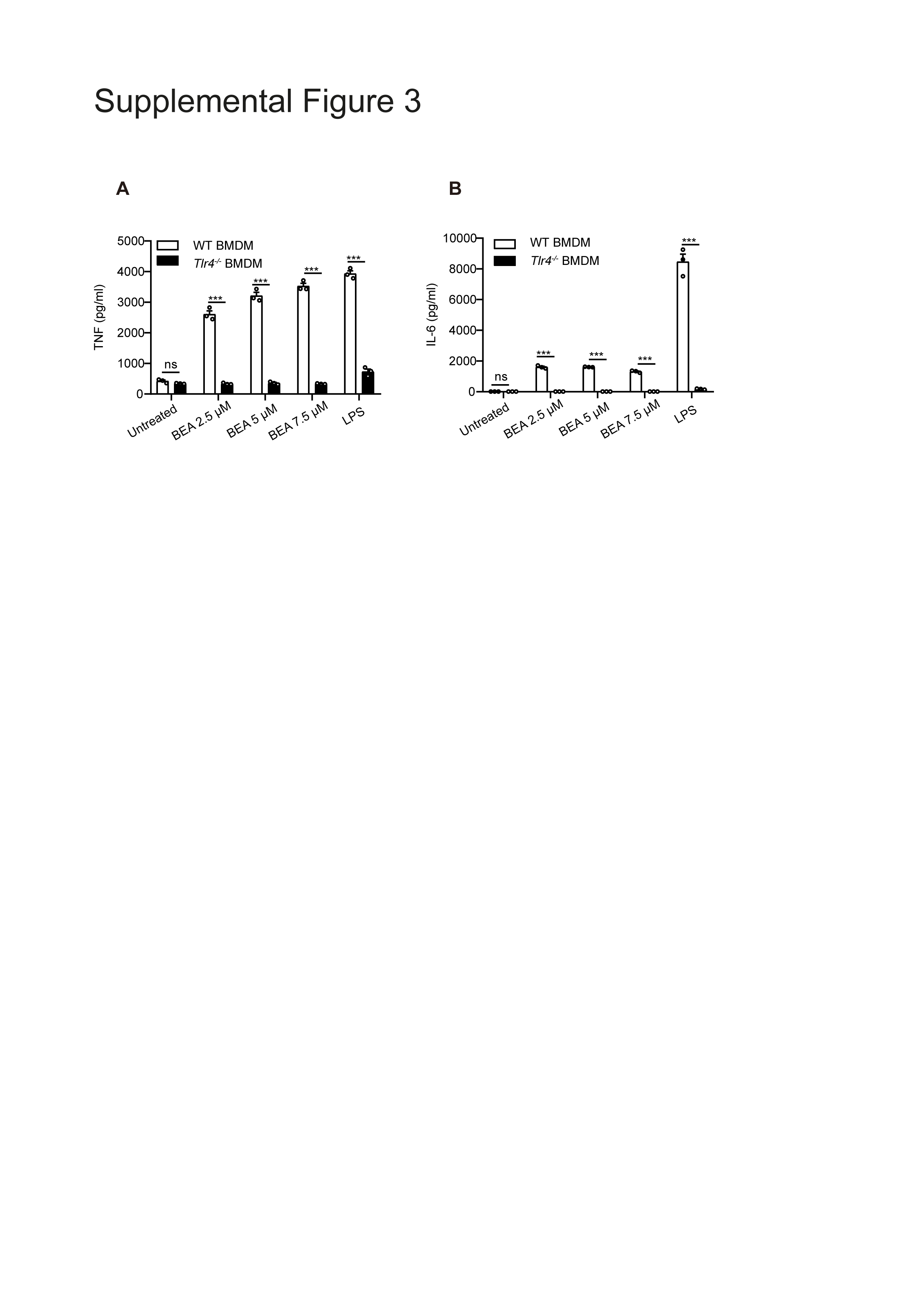

Supplement: Supplementary Figure 3 — Effects of BEA on BMDMs. 1x10^6^ BMDMs derived from WT and Tlr4 -/- mice were stimulated with the indicated concentration of BEA or LPS (100 ng/ml) for 24 hours. TNF (A) and IL-6 (B) expression were analyzed by ELISA. Results shown are representative of two independent experiments using cells from 3 mice per group (n=3). Data in A and B are presented as means ± SEM. Significance was analyzed by Two-way ANOVA with Sidak’s multiple comparisons test. ***p<0.001, ns, no significance. [file Image_3.tif]

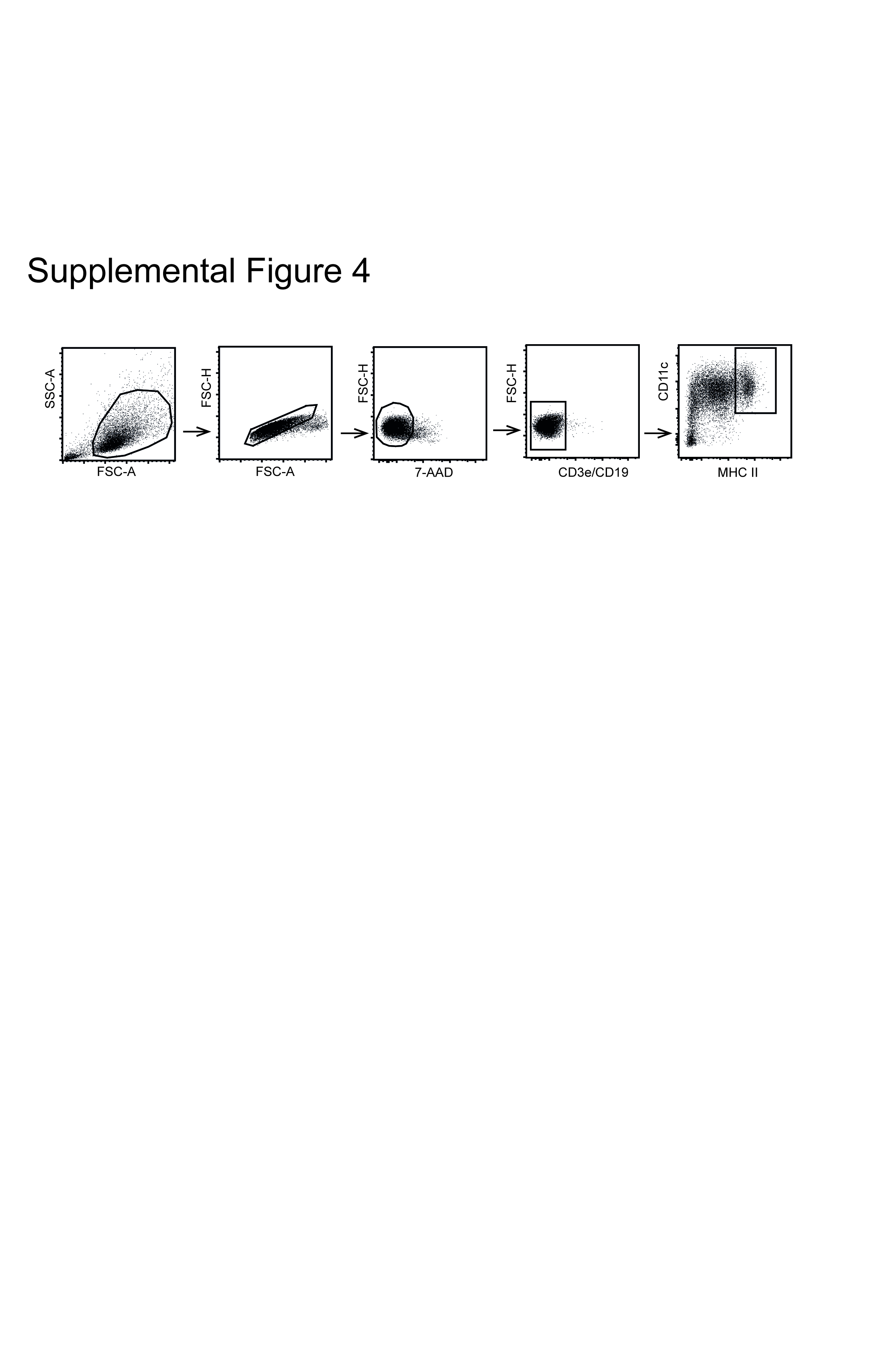

Supplement: Supplementary Figure 4 — Representative gating strategy for cell sorting for RNA seq. Arrows indicate that MHCIIhighCD11c+ cells were sequentially gated from single cells, live cells (7-AAD-), and CD3e-/CD19- cells. Gated MHCIIhighCD11c+ cells were sorted and then stimulated with BEA or LPS. [file Image_4.tif]

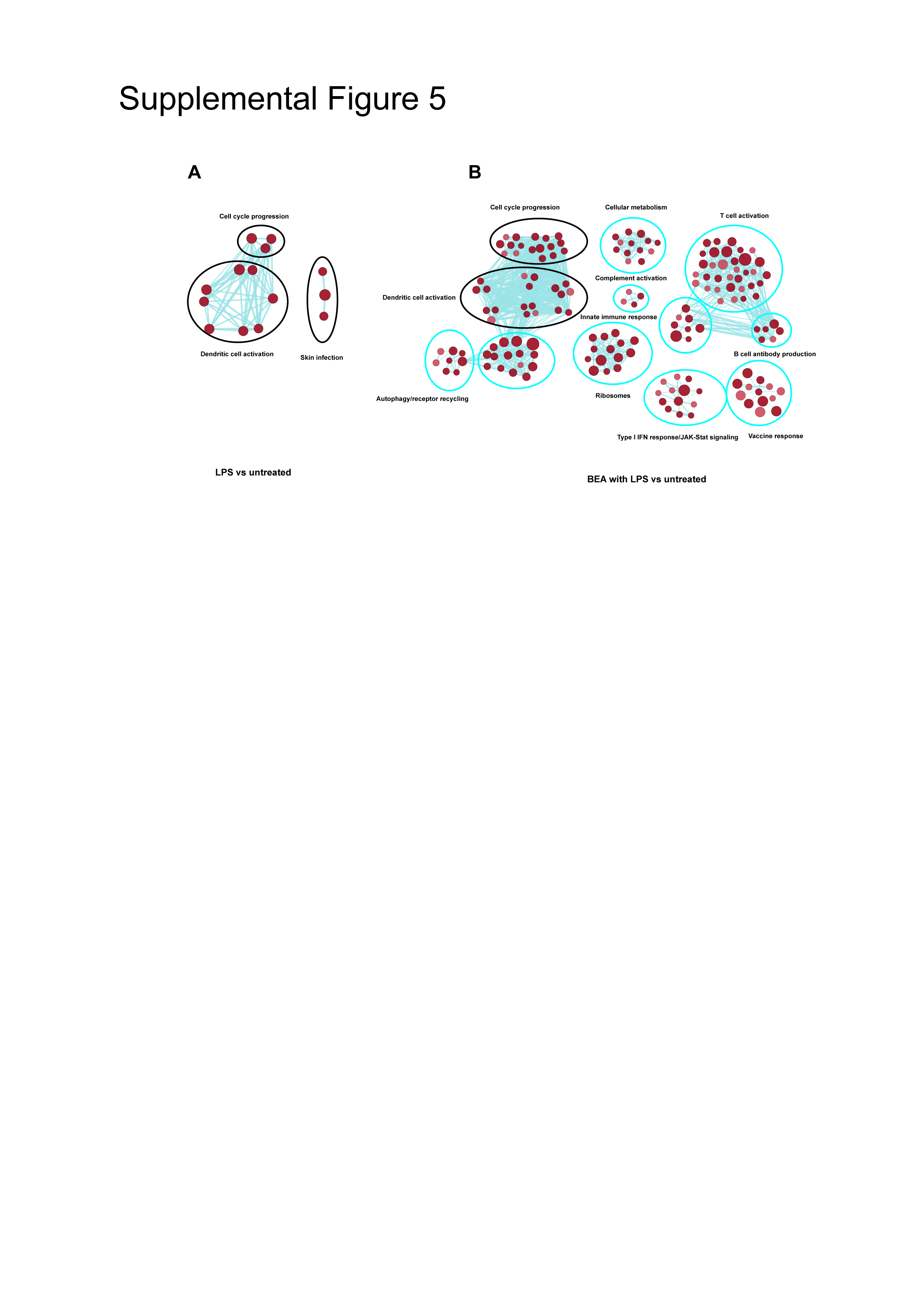

Supplement: Supplementary Figure 5 — Transcriptional changes in BMDCs after treatment with LPS and LPS in combination with BEA. (A) Cytoscape representation of significantly enriched signatures in LPS treated BMDCs compared with untreated BMDCs. (B) Cytoscape representation of significantly enriched signatures in BEA with LPS treated BMDCs compared with untreated BMDCs. [file Image_5.tif]
